# Immunohistochemical Detection of Indoleamine 2,3-Dioxygenase in Spontaneous Mammary Carcinomas of 96 Pet Rabbits

**DOI:** 10.3390/ani14142060

**Published:** 2024-07-13

**Authors:** Sandra Schöniger, Sophie Degner, Claudia Schandelmaier, Heike Aupperle-Lellbach, Qian Zhang, Hans-Ulrich Schildhaus

**Affiliations:** 1 Discovery Life Sciences Biomarker Services GmbH, Germaniastrasse 7, 34119 Kassel, Germany; hans-ulrich.schildhaus@dls.com; 2 Institute of Veterinary Pathology, Faculty of Veterinary Medicine, Leipzig University, An den Tierkliniken 33, 04103 Leipzig, Germany; 3 Laboklin GmbH & Co. KG, Laboratory for Clinical Diagnostics, 97688 Bad Kissingen, Germany; schandelmaier@laboklin.de (C.S.); aupperle@laboklin.de (H.A.-L.); 4 Institute of Pathology, School of Medicine, Technical University of Munich, Trogerstrasse 18, 81675 Munich, Germany; 5 Institute of Anatomy, Experimental Neurobiology, Goethe-University, Theodor-Stern-Kai 7, 60590 Frankfurt, Germany; q.zhang@em.uni-frankfurt.de

**Keywords:** breast cancer research, mammary carcinoma, immunohistochemistry, immuno-oncology, indoleamine 2,3-dioxygenase 1 (IDO1), rabbit, *Oryctolagus cuniculus*, mammary gland, pathology

## Abstract

**Simple Summary:**

Mammary carcinomas have been diagnosed with increasing frequency in pet rabbits. Prognostic biomarkers are limited, and the only available treatment is surgical excision. Additional treatment options are needed, e.g., for animals in which metastases to internal organs preclude complete tumor removal. Human breast cancer may express the immunosuppressive enzyme indoleamine 2,3-dioxygenase 1 (IDO1), that represents a prognostic biomarker and a possible therapeutic target. Since previous studies revealed similarities between human breast cancer and pet rabbit mammary carcinomas, this study investigated IDO1 immunostaining in 96 mammary carcinomas of 96 pet rabbits with an average age of 5.5 years. All rabbits with reported sex were female. Variable percentages of IDO1-positive tumor cells were detected in 90 (94%) carcinomas. Furthermore, IDO1 immunostaining was observed in the secretory epithelial cells of the adjacent non-neoplastic mammary tissue. This study provides further information on the molecular features of mammary carcinomas in rabbits. It also shows similarities in IDO1 expression between rabbit mammary carcinomas and human breast cancer. These findings can have a mutual benefit. They could lead the development of novel treatment options for rabbits with mammary carcinomas. In addition, they further support the value of rabbits with mammary carcinomas for breast cancer research.

**Abstract:**

For mammary carcinomas in pet rabbits, prognostic biomarkers are poorly defined, and treatment is limited to surgical excision. Additional treatment options are needed for rabbit patients for which surgery is not a suitable option. In human breast cancer, the immunosuppressive enzyme indoleamine 2,3-dioxygenase 1 (IDO1) represents a prognostic biomarker and possible therapeutic target. This retrospective immunohistochemical study examined IDO1 in 96 pet rabbit mammary carcinomas with known mitotic count, hormone receptor status, and percentage of stromal tumor infiltrating lymphocytes (TILs). Tumors were obtained from 96 pet rabbits with an average of 5.5 years. All rabbits with reported sex (*n* = 88) were female or female-spayed. Of the carcinomas, 94% expressed IDO1, and 86% had sparse TILs consistent with cold tumors. Statistically significant correlations existed between a higher percentage of IDO1-positive tumor cells, lower mitotic counts, and increased estrogen receptor expression. The threshold for significance was IDO1 staining in >10% of tumor cells. These results lead to the assumption that IDO1 expression contributes to tumorigenesis and may represent a prognostic biomarker and possible therapeutic target also in pet rabbit mammary carcinomas. They also support the value of rabbits for breast cancer research.

## 1. Introduction

In pet rabbits, mammary carcinomas are relatively frequently occurring tumors [1]. Data on possible prognostic biomarkers are limited [2,3,4,5], and the only available treatment is surgical excision [4]. Additional knowledge of the molecular features of pet rabbit mammary carcinomas is required to provide a more concise prognostication of their biological behavior and to reveal novel treatment options. The latter are particularly needed for rabbit patients in which surgery is not a suitable option, e.g., those with a high narcotic risk or with tumor metastases to internal organs that preclude complete surgical removal. Previous studies revealed histologic and immunohistochemical similarities between pet rabbit mammary carcinomas and human breast cancer [2,3,4,6]. The latter may express the enzyme indoleamine 2,3-dioxygenase 1 (IDO1) [7,8,9,10,11,12].

IDO1 is an intracellular heme-containing enzyme of the kynurenine pathway with antimicrobial, immunosuppressive, and tumor-promoting functions [13]. It is localized in the cytoplasm of different cell populations and mediates the breakdown of the essential amino acid L-tryptophan in N-formylkynurenine [13,14]. The latter converts spontaneously to kynurenine, which is further transformed into additional active metabolites. Finally, nicotinamid-adenin-dinucleotid and adenosine triphosphate are generated as the end products of this pathway [14]. The antimicrobial function of IDO1 is caused by tryptophan depletion, which inhibits the growth of viruses, bacteria, and fungal agents [15]. The immunosuppressive action is based on the tissue depletion of tryptophan, which promotes cell cycle arrest and apoptosis in T helper 1 cells as well as the accumulation of tryptophan degradation products that are toxic to lymphocytes and mediate their differentiation into regulatory T cells [13,14]. The latter effect is mediated by the activation of the aryl hydrocarbon receptor (AHR) [13,14].

Previous studies on the tissues of humans and different animal species highlight pathophysiological expression patterns of IDO1 [8,14,16,17,18,19,20,21,22,23].

In people, the IDO1 protein is not only expressed in some normal tissues and organs [8,16,17], but also in malignant tumors of different organ systems [8,14,17]. Within the tumor microenvironment, immunopositive cell populations include tumor cells, macrophages, dendritic cells, lymphocytes, endothelial cells, and stromal cells [8,10,11,14,17,24]. IDO1 expression in tumors, including breast cancer, is often associated with a worse prognosis, since IDO1 and its metabolites inhibit anti-cancer immune defenses and facilitate the survival, motility, and chemoresistance of tumor cells, as well as neo-angiogenesis and metastasis [8,11,12,14,24]. These effects are mediated by the activation of different signaling pathways and receptors including the AHR [12,24]. Therefore, IDO1 has been suggested as a therapeutic target since its inhibition can have a favorable influence on the biological behavior of a tumor as well as its response to established targeted treatment [10,12,24].

Immunostaining for IDO1 has also been observed in the tissue samples of other species, including rabbits [18], mice [19], horses [20], and dogs [21,22,23]. Differences in the tissue expression, however, existed between humans and the different animal species [8,18,21]. Watanabe et al. [18] examined a rabbit duodenum and thyroid gland to find out the cellular location of IDO1. The study of Zhu and Dai [19] reports tissue distribution and cellular location of IDO1 in multiple normal mouse tissues. Schöniger et al. [20] compared IDO1 expression in normal equine endometria and in those affected by inflammatory and degenerative diseases. To the best of the authors’ knowledge, so far, studies on IDO1 expression in animal tumors are only available of dogs. Several canine tumors were examined, including mammary tumors [21], different types of carcinomas [21], sarcomas [21], and melanomas [22,23], as well as multiple normal canine tissues [21]. Similar to human tumors, IDO1 immunopositive cell populations included tumor cells, mononuclear immune cells, and stromal cells [21,22,23]. Of the 14 histologically not-further-classified canine mammary tumors which were included in the study by Ikeda et al. [21], all showed a low IDO1 expression score in tumor cells that was considered as negative and three contained IDO1-positive immune cells [21]. Normal canine mammary tissue was not included in the panel of examined normal tissues [21]. The studies on canine melanocytic tumors included follow-up data and identified IDO1 as a likely prognostic factor in canine melanomas also [22,23].

The aim of this retrospective immunohistochemical study was to examine the expression of IDO1 in pet rabbit mammary carcinomas and adjacent normal tissue to gain knowledge on the pathophysiological expression patterns of this enzyme in rabbits. Furthermore, in the case of positive tumoral IDO1 expression, the subsequent goal was to check for a possible association between IDO1 expression and known histologic, molecular, and immunological tumor data.

Results of this study will provide novel information on the pathophysiological expression of IDO1 in rabbits and the molecular profile of pet rabbit mammary carcinomas. This knowledge can be used in further investigations of the role of this enzyme in normal and diseased mammary tissue of rabbits and is likely also of value for comparative studies of breast cancer in humans and mammary tumors in different animal species.

## 2. Materials and Methods

This retrospective study was performed on tissue sections of 96 mammary carcinomas of 96 pet rabbits, which were stained with hematoxylin–eosin (HE) and immunolabelled for IDO1. The tumors in this study had been included in previous published investigations on estrogen receptor-α (ER-α) and progesterone receptor (PR) expression [2], calponin immunostaining [3], and determination of tumor infiltrating lymphocytes (TILs) [5]. Selected data from these previous studies were included in the present study to examine their possible association with IDO1 expression in pet rabbit mammary carcinomas.

### 2.1. Animals

To obtain information on the pet rabbits with mammary tumors, data on the signalment of the rabbits (sex, age, breed) were extracted from the clinical history.

### 2.2. Tissue Samples

Immediately after their surgical excision in veterinary practices, tissue samples were fixed in 10% neutral buffered formalin and were submitted to a diagnostic laboratory. After 24–48 h of fixation in formalin, the tissue samples were embedded in paraffin wax. All tissue sections used in the present study were prepared within the same laboratory. For this, paraffin blocks were processed routinely, and cut with a microtome (Microm HM400, DiaTec Diagnostische System-Technik, Hallstadt, Germany) into 2 µm sections. The sections were routinely stained with HE. The diagnosis of mammary carcinoma was obtained based on the microscopic examination of the HE-stained sections. Serial sections from each block were prepared immediately prior to immunostaining for IDO1. As all samples were submitted for routine diagnostic purposes and, moreover, were no longer needed for diagnostics, it was not required to submit an animal testing request or to obtain an ethics committee’s approval. This approach is supported by the decision of the local government (RUF-55.2.2-2532-1-86-5).

#### 2.2.1. Histologic Examination

A histologic examination of HE-stained tissue sections was performed to obtain a standardized diagnosis of pet rabbit mammary carcinomas and to compare the histotypes of tumors with and without IDO1 expression.

The classification of the pet rabbit mammary carcinomas, including the determination of histotypes, was performed according to previous published classifications on rabbit mammary tumors [1,2,6] and the 3rd edition of the international histologic classification of mammary tumors of domestic animals [25]. The histotype is defined by the predominant growth pattern(s) [2,25]. In addition, these tumors were examined under consideration of the WHO guidelines for human breast cancer [26].

Furthermore, tumors were examined for the presence of infiltrative or non-infiltrative growth according to the definition of Zappulli et al. [25]. Non-infiltrative tumors are well-demarcated from the adjacent non-neoplastic tissue, whereas infiltrative tumors are characterized by an irregular outline at their periphery [25].

Histopathological examinations were conducted using a Zeiss Axio Scope.A1 microscope (Carl Zeiss AG, Oberkochen, Germany) equipped with 5×, 10×, 20×, and 40× objectives.

#### 2.2.2. Immunohistochemistry for Detection of IDO1

Immunostaining for IDO1 was performed to characterize IDO1 expression in pet rabbit mammary carcinomas and adjacent normal mammary tissue and to correlate results on tumoral IDO expression with available data on histologic and immunological tumor features, hormone receptor status, and calponin expression.

**Figure 1:**
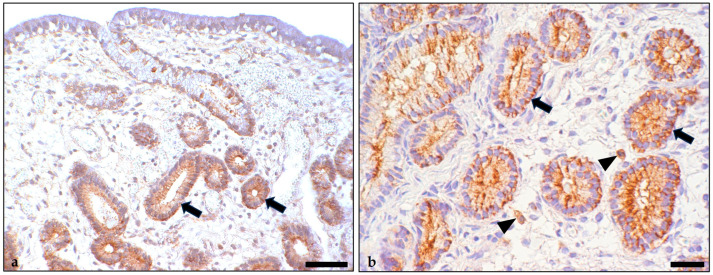
Immunostaining for indoleamine 2,3-dioxygenase 1 in the equine endometrium that served as positive control (**a**,**b**). Immunopositive cell populations were glandular epithelial cells (**a**,**b**: arrows) and scattered mononuclear immune cells morphologically consistent with macrophages (**b**: arrowhead). These showed diffuse cytoplasmic immunolabelling. Bars = 50 µm (**a**), 20 µm (**b**).

Tissue sections from 96 paraffin wax blocks containing formalin-fixed rabbit mammary carcinoma tissue were dewaxed and rehydrated. For blocking of the endogenous peroxidase, tissue sections were placed in methanol containing 3% H_2_O_2_ for 30 min at room temperature (RT). The mouse anti-human IDO1 primary antibody (clone 10.1, Chemicon, Limburg an der Lahn, Germany) was diluted 1:50 in Tris-buffered saline (TBS) with 1% bovine serum albumin. Sections were incubated with the primary antibody overnight at 4 °C. Subsequently, they were thoroughly washed with TBS, followed by applying anti-mouse Dako Envision+ system-HRP polymer (Dako, Hamburg, Germany) for 30 min at RT. After a further washing step, the sections were treated with 3,3′-diaminobenzidine-tetrahydrochloride (DAB) as the chromogen. They were rinsed again and counterstained with Papanicolaou’s solution (Merck, Darmstadt, Germany). As a negative control, the sections were incubated with an isotype-matched nonbinding antibody. Equine endometrium served as positive batch control [20] (Figure 1). The immunostained sections were examined to determine immunopositive cell populations within the normal mammary tissue and the carcinoma and to report cellular location and quality of immunostaining. For this, the 20× to 40× objectives of a Zeiss Axio Scope.A1 microscope were used. Semiquantitative evaluation recorded the percentage of immunopositive tumor cells for each carcinoma using the 20× objective of a Zeiss Axio Scope.A1 microscope.

### 2.3. Data Extracted from Previous Studies

Data from previous studies [2,3,5] were included in this investigation to compare histologic features, hormone receptor status, calponin expression and percentages of TILs between IDO1-positive and IDO1-negative tumors. Furthermore, these data were used for statistical examinations to further characterize pet rabbit mammary carcinomas with and without IDO1 expression.

#### 2.3.1. Histologic Features

The following histologic data were analyzed in detail in a previous study [2] and the results were included in this investigation, i.e., the percent of tumor-associated necrosis, the tumor area with a tubular growth pattern in percent of the entire tumor area [2,27], the mitotic count per ten 40× high power fields (HPFs) under consideration of the field number of the microscope [2,27,28], and the degree of nuclear pleomorphism [2,27]. Tumor-associated necrosis was subdivided into four categories, i.e., minimal, mild, moderate, and marked if it was present in <10%, 10–39%, 40–69%, and >70% of the tumor area, respectively [2]. According to the method of Elston and Ellis [27], each of the following histological parameters, i.e., tubular growth, mitotic count, and nuclear pleomorphism, is evaluated by a numerical scoring system ranging from 1 to 3 points. Obtained points (3–9) are added to obtain the histologic score. The latter is used to calculate the tumor grade, i.e., grade I (histologic scores 3–5), grade II (histologic scores 6–7), and grade III (histologic scores 8–9).

#### 2.3.2. Hormone Receptor and Calponin Expression

Immunostaining for ER-α and PR was described in detail by Degner et al. [2]. The data included in this study are the immunoreactive score (IRS) and the hormone receptor histologic score (H-score) for ER-α and PR. These were determined based on the percentages of immunopositive cells as well as the intensity of immunostaining. The IRS was calculated as [(weakly positive tumor cells) + (moderately positive tumor cells × 5) + (strongly positive tumor cells × 10)/100] [29,30]. The H-score was calculated as (weakly positive cells) + (moderately positive cells × 2) + (strongly positive cells × 3) [28].

Immunolabelling for calponin was described in detail by Degner et al. [3]. The results included in this study are the percentages of calponin immunopositive tumor cells.

#### 2.3.3. Percentages of Tumor-Infiltrating Lymphocytes

The methodical details are described in detail by Schöniger et al. [5]. Analyses of stromal TILs within HE-stained tissue sections were performed according to the guidelines of Salgado et al. [31] and Hendry et al. [32] separately for the central tumor (CT) and the invasive margin (IM). For this the 20× objective of a Zeiss microscope scope A1 with an ocular field number of 23 was used. The 20× field of view corresponds to an area of 1.04 mm^2^. Stromal TILs are defined as the stroma area occupied by lymphocytes and plasma cells in percent over the entire stromal area [31,32]. Stromal TILs were evaluated separately within the CT and the IM [31,32]. The CT represents the tumor area surrounded by the IM. The IM is defined as a 1 mm wide zone at the tumor periphery that is centered at the border of the tumor cell nests with the non-neoplastic host tissue [32]. It is composed of an inner area of tumor tissue (500 µm) and an outer area of peritumoral stroma (500 µm) [32]. The CT and the IM were examined stepwise with the 20× objective [5].

The data included in this study are average and maximal percentages of stromal TILs per 20× objective field of view within the CT and the IM. TILs within the CT were analyzed in all 96 carcinomas. TILs at the IM were examined in 93 cases, since the sections of 3 carcinoma cases did not contain the tumor periphery.

### 2.4. Determination of Immunotypes

The purpose was to categorize pet rabbit mammary carcinomas into known immunotypes and to check possible associations between IDO1 expression and the respective immunotypes.

The classification of rabbit mammary carcinomas into 4 immunotypes, i.e., hot, cold, altered immunosuppressed, and altered-excluded [33], was adjusted to the results of the examinations for TILs in the pet rabbit mammary carcinoma according to Table 1. Determination of immunotype was performed on the sections of the 93 carcinomas that contained CT and IM. Cold tumors were defined by no or only sparse average stromal TILs in the entire tumor area including the CT and the IM. Hot tumors were characterized by abundant stromal TILs, i.e., more than 50% within the CT area and variable percentages of stromal TILs at the IM. The hallmark of altered immunosuppressed tumors was an intermediate percentage of stromal TILs within the CT area without accumulation of stromal TILs at the IM. Altered excluded carcinomas showed an accumulation of average stromal TILs at the IM.

### 2.5. Statistical Analysis

A statistical analysis was conducted to obtain information about if IDO1 expression was statistically associated with tumor features of possible clinical relevance.

For the statistical analysis, the IBM SPSS software version 28 (IBM SPSS Inc., Armonk, NY, USA) was applied.

Using the Pearson correlation coefficient, the percentage of IDO1-positive tumor cells was correlated with the histologic, immunohistochemical, and immunological data that were extracted from previous studies, i.e., the percent of tumor-associated necrosis [2], mitotic count per ten 40× HPFs [2], the degree of nuclear pleomorphism [2], histologic score and grade [2], IRS and H-score for ER-α and PR [2], the percent of calponin-positive tumor cells [3], as well as the average and maximal percentages of stromal TILs per 20× objective field of view within the CT and the IM [5].

A t-test was used for groupwise comparisons of the IDO1-negative tumors (*n* = 6) with tumors containing 1–10% of IDO1-positive tumor cells (*n* = 42) and IDO1-negative tumors (*n* = 6) with tumors containing between 11 and 100 positive tumor cells (*n* = 48), respectively. The following parameters were included in the groupwise comparisons: mitotic count per ten 40× HPFs [2], histologic score and grade [2], as well as IRS and H-score for ER-α and PR [2]. For all applied tests, the significance threshold was set at 0.05.

## 3. Results

### 3.1. Animals

Per clinical history, the pet rabbits in this study were reported as rabbits (*n* = 40), dwarf rabbits (*n* = 30), lion head rabbits (*n* = 3), lion head rabbit mix (*n* = 1), dwarf lop (*n* = 6), lop rabbit (*n* = 3), lop mix (*n* = 1), Angora rabbit (*n* = 1), Teutoburger (*n* = 1), rex (*n* = 1), and as unknown breed (*n* = 9). The ages were not reported in 19 animals. The ages of the remaining rabbits ranged between 1.5 and 10 years (median: 5 years). The sex was not reported for eight rabbits (8%). All rabbits with reported sex were female (*n* = 71; 74%) or female-spayed (*n* = 17; 18%).

### 3.2. Histologic Tumor Classification and IDO1 Expression

#### 3.2.1. Histologic Tumor Classification

**Figure 2:**
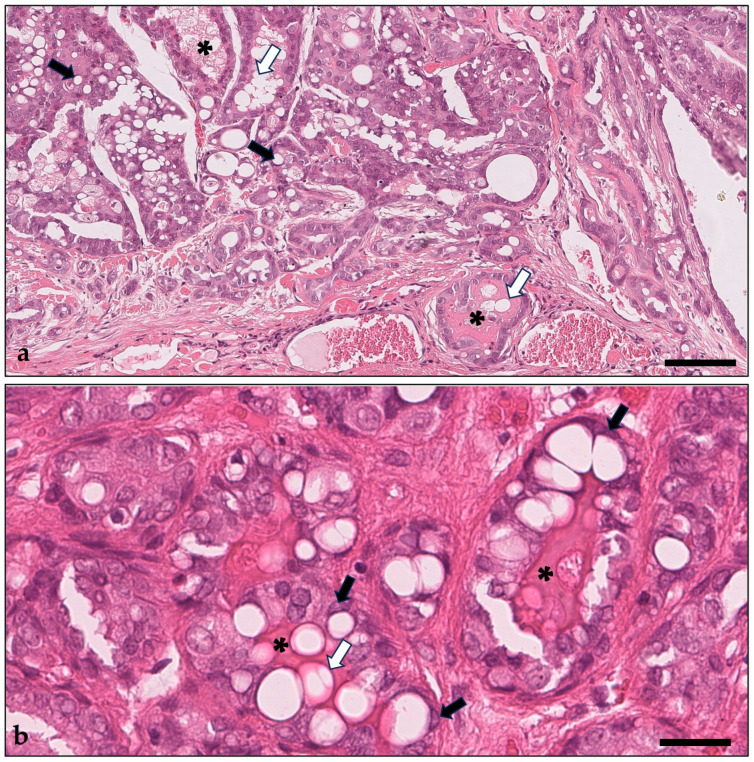
(**a**,**b**) Rabbit mammary carcinoma with secretory activity characterized by proteinaceous fluid in tubular structures (asterisks). Lipid droplets are present in tumor cells (black arrows) and in lumina of tubular structures that also contain proteinaceous material (white arrows). “Cold” immunotype. Overview magnification (**a**) and higher magnification (**b**) showing in detail the presence of lipid droplets in tumor cells (black arrows) and in tubular lumens (white arrows) that also contain proteinaceous material (asterisks). Hematoxylin–eosin stain. Bars = 100 µm (**a**), 30 µm (**b**).

All pet rabbit mammary carcinomas in this study represented simple carcinomas (Figure 2).

In areas with a tubular growth pattern, well-defined tubular structures with distinct lumina are lined by one to two cell layers of neoplastic cells [2,6,25]. The solid growth pattern is characterized by the presence of solid nests and trabecules [2,6,25]. The presence of variably sized cystic spaces covered by one to four layers of neoplastic epithelial cells is consistent with a cystic growth pattern [2,6]. Areas with papillary growth are composed of papillary stromal proliferations lined by two to six layers of tumor cells [2,6]. Areas with a tubulopapillary growth contain tubular structures with intraluminal papillary projections that lined by one to several layers of tumor cells [25].

The tubular histotype dominated, with a percentage of 65% (*n* = 62). Solid (10%, *n* = 10), cystic (10%, *n* = 10), and papillary (5%, *n* = 5) histotypes were also observed. Tumors with the tubulopapillary histotype were uncommon (2%, *n* = 2). In addition, a small number of tumors displayed the presence of two growth patterns at an equal amount of the tumor area, these were diagnosed as combined histotypes, i.e., tubular and solid (5%, *n* = 5), and tubular and cystic (2%, *n* = 2).

Notably, none of the pet rabbit mammary carcinomas would correspond to human tubular carcinoma, since in none of the tumors, more than 90% of the tumor area showed a tubular growth pattern. Using the WHO classification for human breast tumours [26], all pet rabbit mammary carcinomas were consistent with invasive breast carcinoma of no special type (synonym: invasive ductal breast carcinoma).

Infiltrative carcinomas encompassed 71% (68/96) and non-infiltrative tumors encompassed 26% (25/96). In 3% (3/96) tumors, tissue infiltration at the periphery could not be evaluated, since the tumor section contained only the CT area.

All pet rabbit mammary carcinomas showed secretory activity characterized by the accumulation of a proteinaceous material in tubular and cystic structures and/or the detection of lipid droplets in tumor cells and in secretory material (Figure 2). Secretory activity varied from mild (1–10% of the tumor area) to marked (more than 70% of the tumor area).

#### 3.2.2. IDO1 Expression

IDO1 staining in tumor cells was detected in 90 of 96 carcinomas (94%), whereas in six carcinomas, tumor cells were immunonegative.

The six rabbits with IDO1-negative tumors were reported as rabbit (*n* = 1), dwarf rabbit (*n* = 2), and dwarf lop (*n* = 2); in one case, the breed was not included in the clinical history; all rabbits were female. The reported age was 5 (*n* = 1), 6 (*n* = 1) and 7 years (*n* = 2); the ages of two rabbits were not provided in the anamnestic data.

**Figure 3:**
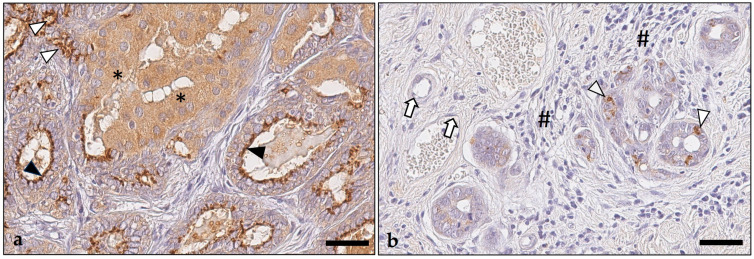
Indoleamine 2,3-dioxygenase 1 immunostaining patterns of pet rabbit mammary carcinomas. (**a**) In this tumor, tumor cells showed a cytoplasmic immunoreaction that was either granular (black and white arrowheads) or nongranular (asterisks). In tumor cells lining tubular structures, the granular stain was restricted to the apical cytoplasm (black arrowheads), whereas tumor cells arranged in small clusters showed a diffuse cytoplasmic granular stain (white arrowheads). The nongranular stain always filled the entire cytoplasm of tumor cells (asterisks). Depicted is the central area of this tumor with an altered-excluded immunotype. (**b**) Tumor with scattered tumor cells displaying granular cytoplasmic staining (white arrowheads). Immune cells (hashtags) and endothelial cells (white arrows) stained negative. Shown is the invasive margin of this tumor with an altered-excluded immunotype. As chromogen, 3,3′-diaminobenzidine-tetrahydrochloride was used. Bars = 100 µm.

**Figure 4:**
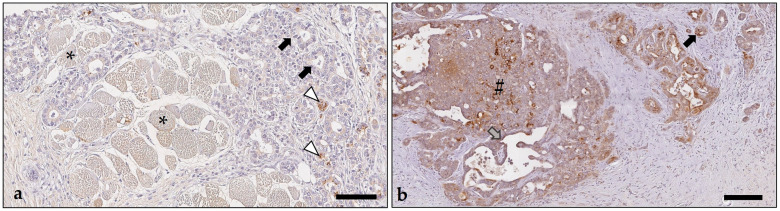
(**a**) Rabbit mammary carcinoma with infiltration of cutaneous trunci muscle. “Cold” immunotype. In this tumor, neoplastic cells infiltrating the skeletal muscle (asterisks) were mainly arranged in a tubular pattern (black arrows) and were predominantly indoleamine 2,3-dioxygenase 1 (IDO1) immunonegative. Only few IDO1-positive cells (white arrowheads) were present. (**b**) Invasive margin of rabbit mammary carcinoma with abundant IDO1 immunopositive tumor cells. These formed solid (hashtags), tubular (black arrow), and cystic structures (gray arrow). “Altered immunosuppressed” immunotype. Bars = 150 µm.

IDO1 was observed in the cytoplasm of the tumor cells as a finely granular or homogeneous nongranular stain (Figure 3 and Figure 4). The immunostaining was either located diffusely within the entire cytoplasm, or it was restricted to the upper apical portion of the cell (Figure 3). Tumor cells with diffuse labeling of the entire cytoplasm were exclusively detected in 77 (80%) tumors, whereas 19 tumors (20%) showed the concurrent presence of tumor cells with apical staining. Diffuse IDO1 expression within the entire cytoplasm of tumor cells was observed in tumor areas with all growth patterns, i.e., solid, tubular, papillary, and cystic, and was either granular or nongranular. In comparison, tumor cells with apical IDO1 staining were restricted to tumor areas with tubular, papillary, or cystic growth; the staining was always granular (Figure 3). Tumor cells with and without IDO1 expression were detected within all tumor areas, including the tumor center and the invasive margin (Figure 4).

**Figure 5:**
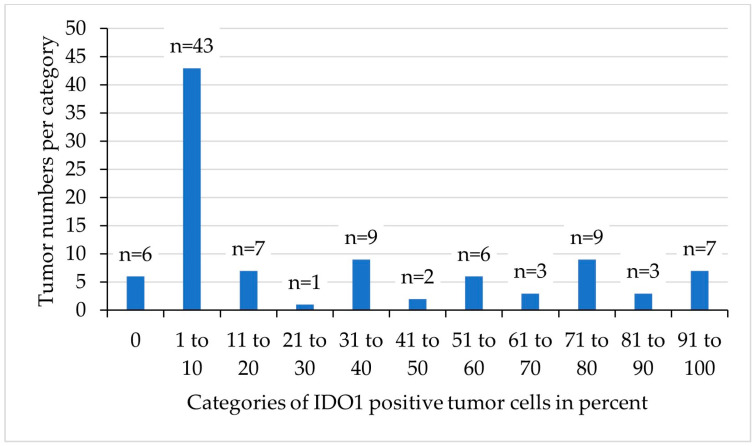
Of the pet rabbit mammary carcinomas, 90 of 96 (94%) contained indoleamine 2,3-dioxygenase 1 (IDO1) immunopositive tumor cells, whereas 6 carcinomas (6%) were immunonegative. In the former, the percentage of IDO1-positive tumor cells was highly variable, ranging from 1% to 100%. Notably, 43 tumors (44%) contained between 1 and 10% positive tumor cells.

**Figure 6:**
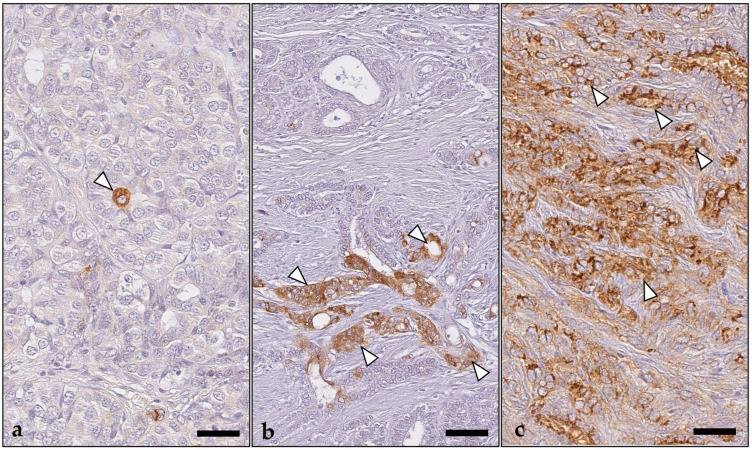
Pet rabbit mammary carcinomas with immunostaining for indoleamine 2,3-dioxygenase 1. The percentages of positive tumor cells were highly variable ranging from very few (**a**, arrowhead) to moderate numbers (**b**, arrowheads) or staining of nearly all neoplastic cells (**c**, arrowheads). Tumors were of “cold” (**a**,**b**) and “altered-excluded” (**c**) immunotypes. Bars = 30 µm.

In the positive carcinomas, the percentage of immunopositive tumor cells was highly variable and ranged from 1 to 100% (median: 10%), whereas 44% of tumors had between 1 and 10% of positive tumor cells (Figure 5 and Figure 6). Notably, none of the examined 96 pet rabbit mammary carcinomas contained IDO1-positive mononuclear immune cells, stromal cells, or endothelial cells (Figure 3 and Figure 6).

**Figure 7:**
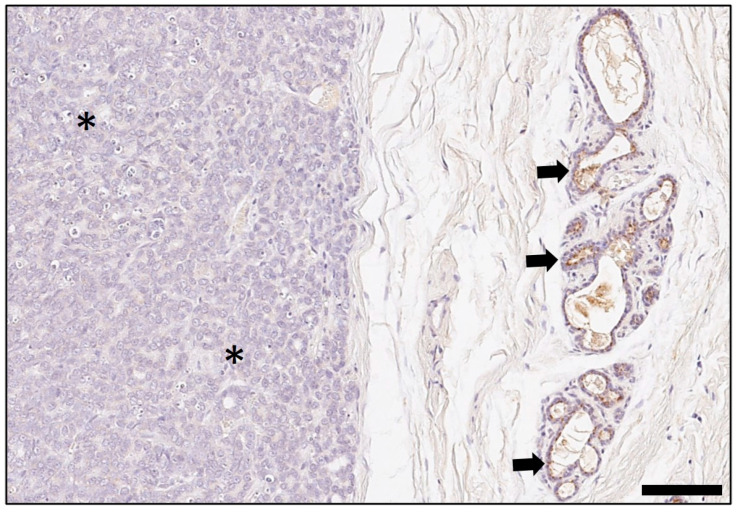
Rabbit mammary carcinoma with negative staining for indoleamine 2,3-dioxygenase 1 in tumor cells (asterisks) and “cold” immunotype, whereas in the adjacent non-neoplastic mammary gland parenchyma, secretory epithelial cells were immunopositive (arrows). As chromogen, 3,3′-diaminobenzidine-tetrahydrochloride was used. Bar = 150 µm.

**Figure 8:**
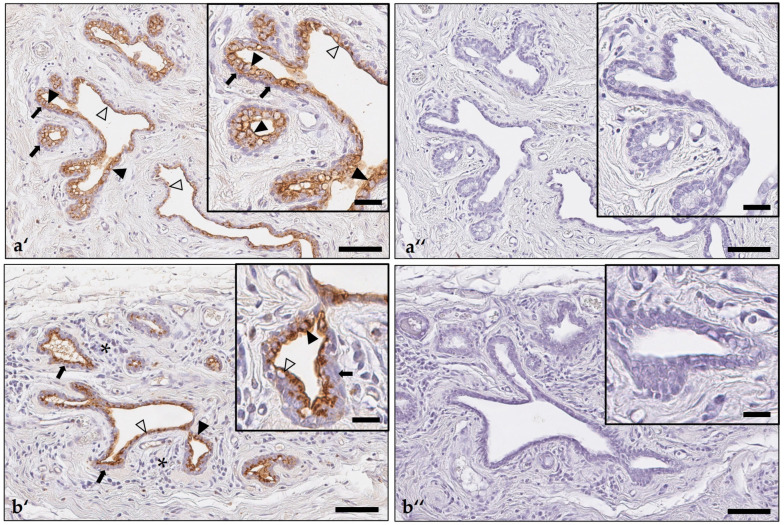
Immunostaining for indoleamine 2,3-dioxygenase 1 in non-neoplastic mammary gland parenchyma without (**a′**) and with (**b′**) moderate periglandular lymphocytic and plasma cellular infiltration. Negative controls in which the primary antibody was replaced by an isotype-matched non-binding antibody (**a″**,**b″**). Non-neoplastic secretory epithelial cells lining tubuloalveolar structures showed cytoplasmic immunostaining, which was observed diffusely within the cytoplasm (black arrowheads), or it was present at the apical pole (gray arrowheads). The negative basal myoepithelial cell layer is labeled by arrows. This is shown in greater detail in the insets (**a′**,**b′**). Periglandular lymphocytes and plasma cells were immunonegative and are marked by asterisks (**b′**). The respective negative controls did not show any labeling, which is shown in higher magnification in the insets (**a″**,**b″**). As chromogen, 3,3′-diaminobenzidine-tetrahydrochloride was used. Bars = 100 µm (**a′**,**a″**,**b′**,**b″**); Bars (insets) = 30 µm (**a′**,**a″**,**b′**,**b″**).

Of the examined 96 excisional biopsies with a mammary carcinoma, 76 (79%) contained adjacent normal/non-neoplastic mammary gland, in which alveoli and intralobular ducts showed positive cytoplasmic immunostaining for IDO1 in 80–100% of lining luminal epithelial cells, whereas the myoepithelial cell layer was negative. The immunosignal was detected diffusely within the cytoplasm, often with apical enhancement, or it was restricted to the apical cytoplasm, it was always granular (Figure 7 and Figure 8).

### 3.3. Characteristics of Carcinomas with and without IDO1 Expression

#### 3.3.1. Histologic Features

There were no apparent differences in the histologic features of carcinomas with or without IDO1 expression in tumor cells.

IDO1-negative carcinomas (*n* = 6): The histotypes were tubular (*n* = 3), tubular and solid (*n* = 2), and solid (*n* = 1). Tumor-associated necrosis encompassed <10% of the tumor area (*n* = 3), 10–39% of the tumor area (*n* = 2), and 40–69% of the tumor area (*n* = 1). The mitotic count was between 4 and 29 mitoses in ten 40× HPFs (median: 7.5). All tumors had moderate nuclear pleomorphism (score 2). One tumor showed a good (grade I) and the remaining tumors a moderate (grade II) differentiation.

IDO1-positive carcinomas (*n* = 90): The histotypes were tubular (*n* = 59; 66%), cystic (*n* = 10; 11%), solid (*n* = 9; 10%), papillary (*n* = 5; 6%), tubular and solid (*n* = 3; 3%), tubulopapillary (*n* = 2; 2%), and tubular and cystic (*n* = 2; 2%). Tumor-associated necrosis was absent (*n* = 22, 24%), encompassed <10% of tumor area (*n* = 40; 44%), 10–39% of tumor area (*n* = 17; 19%), 40–69% of tumor area (*n* = 9; 10%), and ≥70% of the tumor area (*n* = 2; 2%). The mitotic count was between 0 and 30 mitoses in ten 40× HPFs (median: 5). Moderate nuclear pleomorphism was detected in 87 tumors (97%), whereas it was low in 2 (2%) and high in 1 (1%) of the neoplasms. A good (grade I), moderate (grade II), and poor (grade III) differentiation was present in 53 (59%), 35 (39%) and 2 (2%) of the tumors, respectively.

#### 3.3.2. Hormone Receptor and Calponin Expression

Of the examined carcinomas, 62 (65%) were negative for ER-α and PR, whereas 16 (17%) showed immunostaining for both receptors; 16 (17%) were only positive for PR, and 2 (2%) were only positive for ER-α.

The majority of tumors (*n* = 91; 95%) contained calponin-positive tumor cells ranging from 2 to 22 percent (median: 8%), these tumors also included the six IDO1 immunonegative tumors. Tumors with a lack of calponin expression (*n* = 5; 5%) showed a wide range of IDO1 immunostaining in tumor cells, i.e., these neoplasms contained 1, 5, 15, 80, and 100% of IDO1-positive tumor cells, respectively.

IDO1-negative carcinomas (*n* = 6): All IDO1-negative carcinomas were negative for ER-α and PR (IRS = 0, H-score = 0) but contained calponin-positive tumor cells. Their percentages varied between 2% and 19% (median: 8%).

IDO1-positive carcinomas (*n* = 90): Negative immunostaining for ER-α was detected in 72 carcinomas (80%), whereas 18 (20%) stained positive for ER-α. Regarding the latter, the ER-α IRS varied from 0.26 to 2.28 (range: 0–2.28, median: 0), and the ER-α H-score between 14.87 and 124.59 (range: 0–124.59, median: 0). PR staining was absent in 58 neoplasms (65%) and detected in 32 tumors (35%). The PR IRS ranged from 0.16 to 3.77 (range 0–3.77, median: 0). The PR H-score varied from 9.3 to 149.07 (range 0–149.07; median: 0). Five tumors (6%) did not show calponin expression in tumor cells, whereas 85 tumors (94%) contained calponin-positive tumor cells ranging from 2 to 22% (median: 8%).

#### 3.3.3. Percentages of Tumor-Infiltrating Lymphocytes

The maximal and average stromal TILs percentages were highly variable between IDO1-positive and IDO1-negative tumors.

IDO1-negative carcinomas (*n* = 6): The maximal stromal TILs at the invasive margin ranged from 2 to 15% and from 0 to 30% in the central tumor area. Average stromal TILs at the invasive margin varied between 1 and 7% and between 0 and 30% in the central tumor area.

IDO1-positive carcinomas (*n* = 90): Maximal stromal TILs at the invasive margin ranged from 1 to 70% and from 1 to 80% in the central tumor area. Average stromal TILs at the invasive margin varied between 1 and 35% and in the central tumor region, between 1 and 30%. In three tumors, immunotype classification could not be performed due to lack of IM in the examined sections.

### 3.4. Immunotypes

The subclassification into immunotypes (Table 1) could be performed on 93 (87 IDO1-positive and 6 IDO1-negative) tumors. Three tumors did not contain an IM in the examined sections, which precluded the determination of the respective immunotype. The majority of pet rabbit mammary carcinomas represented cold tumors (*n* = 80; 86%), whereas 10% of the tumors (*n* = 9) were consistent with altered immunosuppressed neoplasms and 2% each (*n* = 2) with altered-excluded and hot tumors, respectively. In cold tumors, IDO1 immunostaining of tumor cells varied between 0 and 90% (mean: 31%, SD: 34%), and altered immunosuppressed neoplasms displayed IDO1 labeling in 1–80% of tumor cells (mean: 26%, SD: 30%). The two altered-excluded tumors contained 2% and 60% of IDO1-positive tumor cells, respectively. In hot tumors, 40% and 60% of tumor cells were IDO1 immunopositive. Of the six IDO1-negative tumors, five tumors represented cold tumors, and one was classified as an altered-immunosuppressed tumor. Of the 87 IDO1-positive tumors, 75 (86%) were cold, 8 (9%) altered-immunosuppressed, 2 (2.5%) altered-excluded, and 2 (2.5%) hot.

### 3.5. Results of Statistical Analysis

There was a statistically significant correlation between a higher percentage of IDO1-positive tumor cells and the following parameters, i.e., a lower mitotic count and a rise in IRS and H-scores for ER-α or PR, respectively. No statistically significant correlations existed between the percentage of IDO1-positive tumor cells and the degree of tumor-associated necrosis, nuclear pleomorphism of tumor cells, histologic score or grade, and percentages of calponin-positive tumor cells, as well as maximal and average percentages of stromal TILs in CT and IM (Table 2).

**Figure 9:**
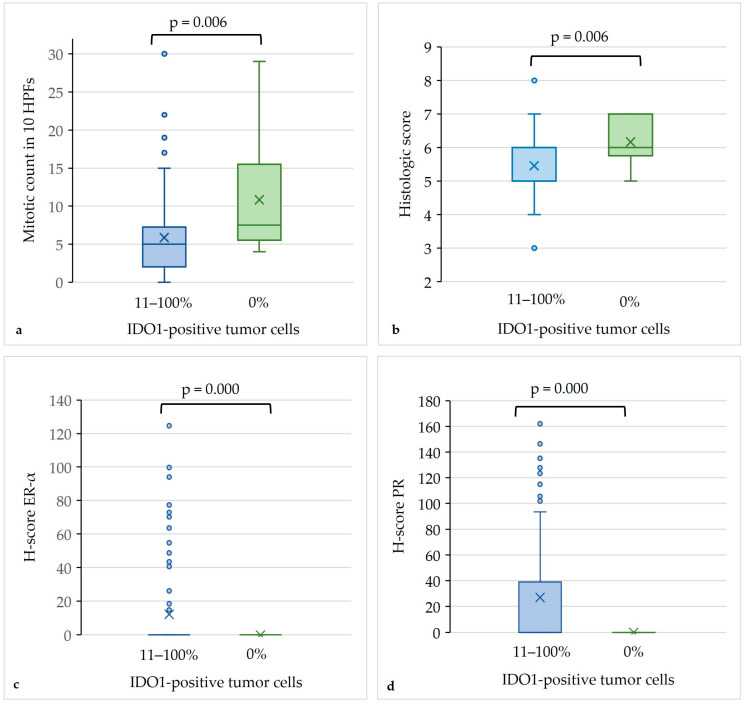
As depicted in the boxplots, statistically significant differences in the mitotic count (**a**), histologic score (**b**), H-score for estrogen receptor-α (ER-α) (**c**), and H-score for progesterone receptor (PR) (**d**) existed between rabbit mammary carcinomas containing 11–100% of tumor cells with immunostaining for indoleamine 2,3-dioxygenase (*n* = 48) and those that were negative (*n* = 6).

As shown in Table 3 and Figure 9, IDO1 expression in more than 10% of tumor cells was determined as the threshold for reaching significance levels for ER-α status and mitotic count between IDO1-positive and IDO1-negative tumors. In detail, the IDO1-negative tumors had significantly higher mitotic counts, higher histologic grades and scores, and reduced ER-α or PR scores than the IDO1-positive tumors with 11–100% positive tumor cells. The comparison of the IDO1-negative tumors with those tumors containing 1–10% IDO1-positive tumor cells did not reach significance levels for the parameters described above, except IRS and H-scores for PR.

## 4. Discussion

This study confirmed that the expression of the IDO1 protein is also present in pet rabbit mammary carcinomas.

### 4.1. IDO1 Staining in Normal/Non-Neoplastic Mammary Gland

To our knowledge, no previously published data on IDO1 expression in rabbit mammary gland tissue exists. The IDO1 protein, however, was detected in epithelial cells of normal rat mammary gland tissue [34], whereas immunostaining for IDO1 in normal human breast tissue was negative [8], and to the authors’ knowledge, no information about its staining in the mammary tissue of other species is available.

IDO1 immunolabelling, however, has also been observed within epithelial cells of the human female genital tract, including surface and glandular epithelial cells in the non-pregnant human endometrium, glandular epithelial cells in the cervix, and epithelial cells in fallopian tubes [8,35]. This led to the hypothesis of intraluminal IDO secretion, which was further supported by the detection of IDO1 activity and kynurenine within cervical mucous [35]. Constitutive IDO1 immunostaining was also observed in surface and/or glandular epithelial cells of the endometrium of primates [36], mice [37], and mares [20]. Based on these findings, it was speculated that the constitutive expression of IDO1 in the female genital tract could modify immune cell activation and may protect against infectious diseases [20,35,36,37].

Similarly, in the mammary gland, IDO1 may contribute—together with other antibacterial factors of the milk—to tissue protection against infection and inflammation [38,39]. This natural protection, however, may be overwhelmed by tissue injury, poor hygienical conditions, and/or immunosuppression [40]. Mastitis frequently occurs in lactating, due to the concurrent presence of several of these predisposing conditions [40].

Based on the detected enhanced apical location and the granularity of the immunostaining, luminal IDO1 secretion may be considered. Notably, IDO1 activity has been detected in cow milk [41]. It was sigificantly higher in the milk of cows with *Prototheca*-induced mastitis than in cows without mastitis [41].

Some antimicrobial factors that are secreted in the rabbit milk are also functional in the intestine of suckling kits [42]. Thus, a contribution of IDO1 to the milk-associated intestinal immune defense may be possible as well.

### 4.2. IDO1 Expression in Rabbit Mammary Carcinomas

A classification of pet rabbit mammary carcinomas into histotypes was performed according to previously published classifications on rabbit mammary tumors [1,2,6] and the 3rd edition of the international histologic classification of mammary tumors of domestic animals [25]. In comparison, the internationally accepted nomenclature for non-proliferative and proliferative lesions in laboratory rabbits solely uses the terminology mammary adenocarcinoma as an umbrella term without further subclassification of different histologic cancer types [43].

In contrast to human breast cancer [8,10,11] and canine mammary tumors [21], examined rabbit mammary carcinomas displayed IDO1 staining only in tumor cells, whereas other tumor-associated cell populations, e.g., mononuclear immune cells and endothelial cells, were negative.

IDO1-positive pet rabbit mammary carcinomas showed two different staining patterns of tumor cells. The non-granular cytoplasmic staining is likely explained by the function of cytoplasmic IDO1, i.e., enzymatic degradation of tryptophane and regulation of cellular signal transduction [44]. The granular cytoplasmic staining pattern suggests IDO1 accumulation within intracellular organelles. In different cell populations, including tumor cells and histiocytic cells, IDO1 is also contained in endosomes [44] and can be secreted in extracellular vesicles [44,45,46]. Thus, in rabbits with mammary carcinomas, IDO1 may also be secreted and could initiate systemic immunosuppressive effects.

Comparable to the findings in examined rabbit tumors, human breast cancer samples displayed a marked variance in the percentage of immunopositive tumor cells, and most IDO1-positive tumors (59%) harbored 1–10% positive tumor cells [10].

In rabbit mammary carcinomas, this study showed a statistically significant correlation between a rise in IDO1 and an increase in ER-α expression. Comparable to this finding, Soliman et al. [7] observed higher IDO1 expression in human ER-α-positive breast cancer compared to ER-α-negative breast cancer.

Increased IDO1 expression in rabbit mammary carcinomas was also significantly associated with a lower mitotic count. This is likely explained by the finding that, in pet rabbit mammary carcinomas, a statistically significant correlation exists between higher ER-α expression and a lower mitotic count as well [2].

In human breast cancer, all molecular subtypes can be IDO1-positive [7,9,10,11], although, the percentages of positive tumors may vary between breast cancer types [10,11]. IDO1 is regarded as an independent unfavorable prognostic marker in multiple cancers [24]. In addition to IDO1, breast cancer may show the concurrent expression of other immunosuppressive and tumor-promoting factors, which can even involve the kynurenine-AHR-axis [10,47].

In this study, results of the groupwise statistical comparisons suggest the existence of at least two different IDO1-associated molecular profiles in rabbit mammary carcinoma, i.e., (1) tumors with IDO1 expression in >10% of tumor cells, higher ER-α status, lower mitotic counts, as well as decreased histological scores and grades and (2) tumors with IDO1 expression in <10% of tumor cells, reduced ER-α levels, higher mitotic counts, as well as increased histological scores and grades. The lower IDO1 expression in rabbit mammary carcinomas with reduced ER-α levels may suggest that, in these tumors, alternative tumor-promoting mechanisms are upregulated. This has to be investigated in future studies.

Previous studies suggested that, in pet rabbit mammary carcinomas, higher percentages of calponin-positive tumor cells [3] and increased percentages of stromal TILs [5] may act as favorable prognostic markers. In addition, statistically significant associations were detected between a higher percentage of calponin-positive tumor cells and higher percentages of stromal TILs [5]. Furthermore, each of these two parameters was associated with a lower mitotic count and a lower histological score [3,5]. The absence of a statistically significant association between these two parameters and IDO1 immunostaining may suggest that IDO1 represents an independent prognostic marker also in pet rabbit mammary carcinomas. This hypothesis would have to be further examined in studies that include rabbit patients with postsurgical follow-up information.

The absence of a statistically significant association between IDO1 levels and percentages of stromal TILs does not rule out an initial or, at least, partial influence of IDO1 on stromal TILs. Since examined sections only represent a snapshot of the findings during tissue selection, IDO1 expression during early tumorigenesis could have contributed to the low number of TILs in most of the examined rabbit mammary carcinomas.

The terms cold and hot tumors are used to describe sparse and abundant infiltration of tumors with lymphocytes and plasma cells, respectively [33]. The designation lymphocyte-rich breast cancer is applied to tumors with more than 50% average stromal TILs [31]. Notably, most rabbit mammary carcinomas had 1–10% stromal TILs, which would warrant their classification as cold tumors.

In neoplastic cells, the expression of IDO1 is induced by an inflamed tumor microenvironment or exists as a constitutive intrinsic feature [24].

In pet rabbit mammary carcinomas, the detected IDO1 expression is regarded as a mainly constitutive event due to the presence of only sparse immune cell infiltrates in most tumors. Since both hot tumors of this study showed IDO1 immunostaining in higher numbers of tumor cells, i.e., 40% and 60% of tumor cells, an additional inflammation mediated IDO1 upregulation should be considered. One of the two altered-excluded tumors displayed IDO1 expression in 60% of tumor cells, which could have caused or at least contributed to immune cell exclusion from the main tumor area. Since the other altered-excluded carcinoma contained 2% IDO1-positive tumor cells, the existence of additional immunosuppressive mechanisms within the tumor area appears likely. Similarly, also in the altered-immunosuppressive carcinomas additional factors of immunosuppression appear likely due to the wide range of IDO1-positive tumor cells.

### 4.3. Mutual Benefit of the Results for Rabbit Patients and Breast Cancer Research

This study provided novel molecular data on pet rabbit carcinomas, which can serve as the basis for future investigations into their clinical relevance. The observation that most rabbit mammary carcinomas were IDO1-positive and showed a cold tumor status may suggest that IDO1 could be involved in impacting anti-cancer immune defenses also in pet rabbit mammary carcinomas. This assumption, however, shall be treated with caution, since only 2% of the examined tumors were hot tumors.

Current treatment concepts for cold tumors aim to convert them into hot tumors by increasing their antigenicity and immunogenicity [48]. Thus, further studies are necessary to reveal if IDO1 inhibition in pet rabbit mammary carcinomas will stimulate immune cell infiltration within the tumor area.

Tumoral IDO1 expression, however, not only inhibits anti-cancer immune defenses but also facilitates the survival, motility, and chemoresistance of tumor cells, as well as neo-angiogenesis and metastasis [11,12,14,24]. The unfavorable prognosis of IDO1-positive cancers is likely often attributed to the combined influence of several of the IDO1-induced processes [14,24].

Due to the selective expression of IDO1 in tumor cells, IDO1 inhibition in rabbit mammary carcinomas may have the benefit of directly targeting tumor cells without causing possible interfering effects also in other cell populations. Since IDO1 is expressed in normal pet rabbit mammary tissue, systemic administration of an inhibitor of the IDO1 pathway could potentially lead to local site effects, which could be avoided by selecting therapeutic modes that selectively target the tumor tissue, such as antibody drug conjugates [49].

Breast cancer is the most common cancer type in women. Rabbits have been suggested as animal models for human breast cancer, since they fulfill numerous criteria for an ideal animal model [4]. In human breast cancer, IDO1 expression has been reported in all molecular subtypes [7,9,10]. Certain ER-α-negative breast cancer types, i.e., triple-negative breast cancer, and cold tumors have only limited treatment options [50,51,52]. For this type of hormone receptor negative breast cancer, animal models with spontaneous mammary carcinomas would be most valuable, and the majority of pet rabbit mammary carcinomas represent IDO1-positive, ER-α-negative, and cold tumors.

In clinical trials, it has been shown that inhibition of IDO1 in different human tumors does not always yield the expected clinical benefit [47,53]. A recent investigation identified indole 3-pyruvate (I3P), a tryptophane metabolite created by the enzyme interleukin 4-induced 1 (IL4I1), as an additional AHR ligand and driver of tumor growth in hepatocellular carcinoma [53]. Furthermore, it has been shown that breast cancer may express not only IDO1, but also IL4I1 [53]. This suggests that activation of the tryptophane–IL4I1–I3P pathway could possibly contribute to breast cancer tumorigenesis as well.

Further studies are necessary to find out if the tryptophane–IL4I1–I3P pathway also exists in mammary carcinomas of pet rabbits. Results of these investigations would further assist in the development of effective, novel therapeutic options for pet rabbit mammary carcinomas. They also would help to define the areas in which pet rabbit mammary carcinomas could best support breast cancer research.

## 5. Conclusions

This study reveals novel molecular data on spontaneous pet rabbit mammary carcinomas. This will likely serve as baseline for future studies into novel treatment options of pet rabbit mammary carcinomas. In addition, it likely improves the value of rabbit mammary carcinomas for breast cancer research.

## Figures and Tables

**Table 1:** Classification of 93 pet rabbit mammary carcinomas into 4 immunotypes.

Tumor Classification into Immunotypes	Average Stromal TILs in Central Tumor	Average Stromal TILs at Invasive Margin
Hot	>50–100%	0–100%
Cold	0–10%	0–10%
Altered immunosuppressed	>10–50%	0–50%
Altered excluded	0–10%	>10–100%

TILs = Tumor-infiltrating lymphocytes defined as lymphocytes and plasma cells.

**Table 2:** Results of analyzed statistical correlations.

Parameter 1	Parameter 2	Correlation	Cases
% IDO1 positive TCs	**Mitotic count**	***p* = 0.007; r = −0.272**	*n* = 96
	**IRS ER-α**	***p* = 0.000; r = 0.397**	*n* = 96
	**H-score ER-α**	***p* = 0.000; r = 0.437**	*n* = 96
	**IRS PR**	***p* = 0.000; r = 0.476**	*n* = 96
	**H-score PR**	***p* = 0.000; r = 0.510**	*n* = 96
	Tumor-associated necrosis	*p* = 0.082; r = −0.178	*n* = 96
	Nuclear pleomorphism	*p* = 0.519; r = 0.067	*n* = 96
	Histologic score	*p* = 0.093; r = −0.172	*n* = 96
	Histologic grade	*p* = 0.123; r = −0.158	*n* = 96
	Calponin-positive TCs (%)	*p* = 0.668; r = 0.044	*n* = 96
	Max. stromal TILs CT	*p* = 0.538; r = 0.064	*n* = 96
	Average stromal TILs CT	*p* = 0.308; r = 0.105	*n* = 96
	Max. stromal TILs IM	*p* = 0.333; r = 0.102	*n* = 93
	Average stromal TILs IM	*p* = 0.624; r = 0.052	*n* = 93

Correlations with statistically significant results are indicated in bold letters. IDO1 = indoleamine 2,3-dioxygenase 1; % = percent; TCs = tumor cells; ER-α = estrogen receptor-α; PR = progesterone receptor; IRS = immunoreactive score [29,30]; H-score = hormone receptor histologic score [28], Mitotic count = number of mitotic cells in 10 HPFs under consideration of the field number of the microscope [2,27]; tumor-associated necrosis: necrosis in percent of the tumor area; Nuclear pleomorphism [2,27]; Histologic score [2,27]; Histologic grade [2,27]; Max. or average stromal TILs CT = maximal or average percentages of stromal tumor infiltrating lymphocytes within a 20× field of view in the central tumor [5,31,32]; Max. or average stromal TILs IM = maximal or average percentages of stromal tumor infiltrating lymphocytes within a 20× field of view in the central tumor [5,31,32]; *p* = *p*-value (statistical significance set at 0.05); r = Pearson correlation coefficient.

**Table 3:** Groupwise comparison of IDO1-negative tumors with those tumors expressing IDO1 in 1–10% (**a**) and 11–100% of tumor cells (**b**), respectively.

(**a**)			
**Parameter**	**IDO1 Neg.** **(MW ± SD)**	**IDO1 1–10% TCs** **(MW ± SD)**	***p*-Value**
Mitotic count	10.83 ± 9.20	7.12 ± 6.46	0.378
Histologic score	6.17 ± 0.75	5.57 ± 1.17	0.130
Tumor grade	1.83 ± 0.41	1.48 ± 0.59	0.096
ER-α IRS	0.00 ± 0.00	0.03 ± 0.11	0.084
ER-α H-score	0.00 ± 0.00	2.44 ± 9.42	0.100
PR IRS	0.00 ± 0.00	0.24 ± 0.65	0.022
PR H-score	0.00 ± 0.00	11.02 ± 9.18	0.019
(**b**)			
**Parameter**	**IDO1 Neg.** **(MW ± SD)**	**IDO1 11–100% TCs** **(MW ± SD)**	***p*-Value**
Mitotic count	10.83 ± 9.20	4.75 ± 4.19	0.006
Histologic score	6.17 ± 0.75	5.35 ± 0.98	0.006
Tumor grade	1.83 ± 0.41	1.40 ± 0.49	0.047
ER-α IRS	0.00 ± 0.00	0.30 ± 0.57	0.001
ER-α H-score	0.00 ± 0.00	20.74 ± 35.58	0.000
PR IRS	0.00 ± 0.00	0.90 ± 1.26	0.000
PR H-score	0.00 ± 0.00	41.04 ± 53.45	0.000

IDO1 = indoleamine 2,3 dioxygenase 1; MW = mean value; SD = standard deviation; neg. = negative; % = percent; TCs = tumor cells; MW = mean value; SD = standard deviation; ER-α = estrogen receptor-α; PR = progesterone receptor; IRS = immunoreactive score; H-score = hormone receptor histologic score.

## Data Availability

Further information on the data included in this study is available from the corresponding author upon reasonable request.

## References

[1] Baum B., Hewicker-Trautwein M. (2015). Classification and epidemiology of mammary tumours in pet rabbits (*Oryctolagus cuniculus*). J. Comp. Pathol..

[2] Degner S., Schoon H.A., Laik-Schandelmaier C., Aupperle-Lellbach H., Schöniger S. (2018). Estrogen receptor-α and progesterone receptor expression in mammary proliferative lesions of female pet rabbits. Vet. Pathol..

[3] Degner S., Schoon H.A., Degner S., Baudis M., Schandelmaier C., Aupperle-Lellbach H., Schöniger S. (2019). Expression of myoepithelial markers in mammary carcinomas of 119 pet rabbits. Animals.

[4] Schöniger S., Degner S., Jasani B., Schoon H.A. (2019). A review on mammary tumors in rabbits: Translation of pathology into medical care. Animals.

[5] Schöniger S., Degner S., Zhang Q., Schandelmaier C., Aupperle-Lellbach H., Jasani B., Schoon H.-A. (2020). Tumor infiltrating lymphocytes in pet rabbit mammary carcinomas: A study with relevance to comparative pathology. Animals.

[6] Schöniger S., Horn L.C., Schoon H.-A. (2014). Tumors and tumor-like lesions in the mammary gland of 24 pet rabbits: A histomorphological and immunohistochemical characterization. Vet. Pathol..

[7] Soliman H., Rawal B., Fulp J., Lee J.H., Lopez A., Bui M.M., Khalil F., Antonia S., Yfantis H.G., Lee D.H. (2013). Analysis of indoleamine 2-dioxygenase (IDO1) expression in breast cancer by immunohistochemistry. Cancer Immunol. Immunother..

[8] Théate I., van Baren N., Pilotte L., Moulin P., Larrieu P., Renauld J.C., Hervé C., Gutierrez-Roelens I., Marbaix E., Sempoux C. (2015). Extensive profiling of the expression of indoleamine 2,3 dioxygenase 1 protein in normal and tumoral human tissues. Cancer Immunol. Res..

[9] Carvajal-Hausdorf D.E., Mani N., Velcheti V., Schalper K.A., Rimm D.L. (2017). Objective measurement and clinical significance of IDO1 protein in hormone receptor-positive breast cancer. J. Immunother. Cancer.

[10] Dill E.A., Dillon P.M., Bullock T.N., Mills A.M. (2018). IDO expression in breast cancer: An assessment of 281 primary and metastatic cases with comparison to PD-L1. Mod. Pathol..

[11] Wei L., Zhu S., Li M., Li F., Wei F., Liu J., Ren X. (2018). High indoleamine 2,3-dioxygenase is correlated with microvessel density and worse prognosis in breast cancer. Front. Immunol..

[12] Zhao X., Jiang Y., Xu M., Hu J., Feng N., Deng H., Lu C., Huang T. (2022). Indoleamine 2,3-dioxygenase 1 regulates breast cancer tamoxifen resistance through interleukin-6/signal transducer and activator of transcription. Toxicol. Appl. Pharmacol..

[13] Bilir C., Sarisozen C. (2017). Indoleamine 2,3-dioxygenase (IDO): Only an enzyme or a checkpoint controller?. J. Oncol. Sci..

[14] Hornyák L., Dobos N., Koncz G., Karányi Z., Páll D., Szabó Z., Halmos G., Székvölgyi L. (2018). The role of indoleamine-2,3-dioxygenase in cancer development, diagnostics, and therapy. Front. Immunol..

[15] Curti A., Trabanelli S., Salvestrini V., Baccarani M., Lemoli R.M. (2009). The role of indoleamine 2,3-dioxygenase in the induction of immune tolerance: Focus on hematology. Blood.

[16] Yamazaki F., Kuroiwa T., Takikawa O., Kido R. (1985). Human indolylamine 2,3-dioxygenase: Its tissue distribution, and characterization of the placental enzyme. Biochem. J..

[17] Vigneron N., van Baren N., Van den Eynde B.J. (2015). Expression profile of the human IDO1 protein, a cancer drug target involved in tumoral immune resistance. Oncoimmunology.

[18] Watanabe Y., Yoshida R., Sono M., Hayaishi O. (1981). Immunohistochemical localization of indoleamine 2,3-dioxygenase in the argyrophilic cells of rabbit duodenum and thyroid gland. J. Histochem. Cytochem..

[19] Dai X., Zhu B.T. (2010). Indoleamine 2,3-dioxygenase tissue distribution and cellular localization in mice: Implications for its biological functions. J. Histochem. Cytochem..

[20] Schöniger S., Gräfe H., Richter F., Schoon H.A. (2018). Expression of indoleamine 2,3-dioxygenase 1 as transcript and protein in the healthy and diseased equine endometrium. Res. Vet. Sci..

[21] Ikeda N., Kato D., Tsuboi M., Yoshitake R., Eto S., Yoshimoto S., Shinada M., Kamoto S., Hashimoto Y., Takahashi Y. (2021). Detection of indoleamine 2,3-dioxygenase 1-expressing cells in canine normal and tumor tissues. J. Vet. Med. Sci..

[22] Porcellato I., Brachelente C., De Paolis L., Menchetti L., Silvestri S., Sforna M., Vichi G., Iussich S., Mechelli L. (2019). FoxP3 and IDO in canine melanocytic tumors. Vet. Pathol..

[23] Porcellato I., Brachelente C., Cappelli K., Menchetti L., Silvestri S., Sforna M., Mecocci S., Iussich S., Leonardi L., Mechelli L. (2021). FoxP3, CTLA-4, and IDO in canine melanocytic tumors. Vet. Pathol..

[24] Meireson A., Devos M., Brochez L. (2020). IDO expression in cancer: Different compartment, different functionality?. Front. Immunol..

[25] Zappuli V., Peňa L., Rasotto R., Goldschmidt M.H., Gama A., Scruggs J.L., Kiupel M., Kiupel M. (2019). Mammary tumors. Surgical Pathology of Tumors of Domestic Animals.

[26] WHO Classification of Tumours Editorial Board (2019). Breast Tumours.

[27] Elston C.W., Ellis I.O. (1991). Pathological prognostic factors in breast cancer. I. The value of histologic grade in breast cancer: Experience from a large study with long-term follow-up. Histopathology.

[28] Ellis I.O., Pinder S.E., Lee A.H.S., Fletcher C.D.M. (2007). Tumors of the breast. Diagnostic Histopathology of Tumors.

[29] Remmele W., Stegner H.E. (1987). Vorschlag zur einheitlichen Definition eines Immunreaktiven Score (IRS) für den immunhistochemischen Ostrogenrezeptor-Nachweis (ER-ICA) im Mammakarzinomgewebe [Recommendation for uniform definition of an immunoreactive score (IRS) for immunohistochemical estrogen receptor detection (ER-ICA) in breast cancer tissue]. Pathologe.

[30] Aupperle H., Özgen S., Schoon H.A., Schoon D., Hoppen H.O., Sieme H., Tannapfel A. (2000). Cyclical endometrial steroid hormone receptor expression and proliferation intensity in the mare. Equine Vet. J..

[31] Salgado R., Denkert C., Demaria S., Sirtaine N., Klauschen F., Pruneri G., Wienert S., Van den Eynden G., Baehner F.L., Pénault-Llorca F. (2015). The evaluation of tumor-infiltrating lymphocytes (TILs) in breast cancer: Recommendations by an international TILs working group 2014. Ann. Oncol..

[32] Hendry S., Salgado R., Gevaert T., Russell P.A., John T., Thapa B., Christie M., Van De Vijver K., Estrada M.V., Gonzalez-Ericsson P.I. (2017). Assessing tumor-infiltrating lymphocytes in solid tumors: A practical review for pathologists and proposal for a standardized method from the international immunooncology biomarkers working group: Part 1: Assessing the host immune response, TILs in invasive breast carcinoma and ductal carcinoma in situ, metastatic tumor deposits and areas for further research. Adv. Anat. Pathol..

[33] Galon J., Bruni D. (2019). Approaches to treat immune hot, altered and cold tumours with combination immunotherapies. Nat. Rev. Drug Discov..

[34] Hill M., Pereira V., Chauveau C., Zagani R., Remy S., Tesson L., Mazal D., Ubillos L., Brion R., Ashgar K. (2005). Heme oxygenase-1 inhibits rat and human breast cancer cell proliferation: Mutual cross inhibition with indoleamine 2,3-dioxygenase. Faseb J..

[35] Sedlmayr P., Blaschitz A., Wintersteiger R., Semlitsch M., Hammer A., MacKenzie C.R., Walcher W., Reich O., Takikawa O., Dohr G. (2002). Localization of indoleamine 2,3-dioxygenase in human female reproductive organs and the placenta. Mol. Hum. Reprod..

[36] Drenzek J.G., Breburda E.E., Burleigh D.W., Bondarenko G.I., Grendell R.L., Golos T.G. (2008). Expression of indoleamine 2,3-dioxygenase in the rhesus monkey and common marmoset. J. Reprod. Immunol..

[37] Jeddi-Tehrani M., Abbasi N., Dokouhaki P., Ghasemi J., Rezania S., Ostadkarampour M., Rabbani H., Akhondi M.A., Fard Z.T., Zarnani A.H. (2009). Indoleamine 2,3- dioxygenase is expressed in the endometrium of cycling mice throughout the oestrous cycle. J. Reprod. Immunol..

[38] Eker F., Akdaşçi E., Duman H., Yalçıntaş Y.M., Canbolat A.A., Kalkan A.E., Karav S., Šamec D. (2024). Antimicrobial properties of Colostrum and Milk. Antibiotics.

[39] Maertens L., Lebas F., Szendrö Z. (2006). Rabbit milk: A review of quantity, quality and non-dietary affecting factors. World Rabbit Sci..

[40] Vasiu I., Wochnik M., Dąbrowski R. (2023). Mammary gland inflammation in rabbits does (*Oryctolagus cuniculus*): A systematic review. Reprod Domest Anim..

[41] Bochniarz M., Piech T., Kocki T., Iskra M., Krukowski H., Jagielski T. (2021). Tryptophan, kynurenine and kynurenic acid concentrations in milk and serum of dairy cows with Prototheca mastitis. Animals.

[42] Gallois M., Gidenne T., Tasca C., Caubet C., Coudert C., Milon A., Boullier S. (2007). Maternal milk contains antimicrobial factors that protect young rabbits from enteropathogenic Escherichia coli infection. Clin. Vaccine Immunol..

[43] Bradley A.E., Wancket L.M., Rinke M., Gruebbel M.M., Saladino B.H., Schafer K., Katsuta O., Garcia B., Chanut F., Hughes K. (2021). International harmonization of nomenclature and diagnostic criteria (INHAND): Nonproliferative and proliferative lesions of the rabbit. J. Toxicol. Pathol..

[44] Pallotta M.T., Rossini S., Suvieri C., Coletti A., Orabona C., Macchiarulo A., Volpi C., Grohmann U. (2022). Indoleamine 2,3-dioxygenase 1 (IDO1): An up-to-date overview of an eclectic immunoregulatory enzyme. Febs J..

[45] Pezzicoli G., Tucci M., Lovero D., Silvestris F., Porta C., Mannavola F. (2020). Large extracellular vesicles—A new frontier of liquid biopsy in oncology. Int. J. Mol. Sci..

[46] Hosseini R., Asef-Kabiri L., Yousefi H., Sarvnaz H., Salehi M., Akbari M.E., Eskandari N. (2021). The roles of tumor-derived exosomes in altered differentiation, maturation and function of dendritic cells. Mol. Cancer.

[47] Sadik A., Patterson L.F.S., Öztürk S., Mohapatra S.R., Panitz V., Secker P.F., Pfänder P., Loth S., Salem H., Prentzell M.T. (2020). IL4I1 is a metabolic immune checkpoint that activates the AHR and promotes tumor progression. Cell.

[48] Prendergast G.C., Mondal A., Dey S., Laury-Kleintop L.D., Muller A.J. (2018). Inflammatory reprogramming with IDO1 inhibitors: Turning immunologically unresponsive ‘cold’ tumors ‘hot’. Trends Cancer.

[49] Jin S., Sun Y., Liang X., Gu X., Ning J., Xu Y., Chen S., Pan L. (2022). Emerging new therapeutic antibody derivatives for cancer treatment. Signal Transduct. Target. Ther..

[50] Bonaventura P., Shekarian T., Alcazer V., Valladeau-Guilemond J., Valsesia-Wittmann S., Amigorena S., Caux C., Depil S. (2019). Cold tumors: A therapeutic challenge for immunotherapy. Front. Immunol..

[51] Geurts V., Kok M. (2023). Immunotherapy for metastatic triple negative breast cancer: Current paradigm and future approaches. Curr. Treat. Options Oncol..

[52] Li L., Zhang F., Liu Z., Fan Z. (2023). Immunotherapy for triple-negative breast cancer: Combination strategies to improve outcome. Cancers.

[53] Venkateswaran N., Garcia R., Lafita-Navarro M.C., Hao Y.H., Perez-Castro L., Nogueira P.A.S., Solmonson A., Mender I., Kilgore J.A., Fang S. (2024). Tryptophan fuels MYC-dependent liver tumorigenesis through indole 3-pyruvate synthesis. Nat. Commun..

